# Reduced expression of CDP-DAG synthase changes lipid composition and leads to male sterility in *Drosophila*

**DOI:** 10.1098/rsob.150169

**Published:** 2016-01-20

**Authors:** Barbara Laurinyecz, Mária Péter, Viktor Vedelek, Attila L. Kovács, Gábor Juhász, Péter Maróy, László Vígh, Gábor Balogh, Rita Sinka

**Affiliations:** 1 Department of Genetics, University of Szeged, Szeged, Hungary; 2 Institute of Biochemistry, Biological Research Centre of the Hungarian Academy of Sciences, Szeged, Hungary; 3 Department of Anatomy, Eötvös Loránd University, Budapest, Hungary

**Keywords:** *Drosophila*, spermatogenesis, lipid

## Abstract

*Drosophila* spermatogenesis is an ideal system to study the effects of changes in lipid composition, because spermatid elongation and individualization requires extensive membrane biosynthesis and remodelling. The bulk of transcriptional activity is completed with the entry of cysts into meiotic division, which makes post-meiotic stages of spermatogenesis very sensitive to even a small reduction in gene products. In this study, we describe the effect of changes in lipid composition during spermatogenesis using a hypomorphic male sterile allele of the *Drosophila CDP-DAG synthase* (*CdsA*) gene. We find that the *CdsA* mutant shows defects in spermatid individualization and enlargement of mitochondria and the axonemal sheath of the spermatids. Furthermore, we could genetically rescue the male sterile phenotype by overexpressing Phosphatidylinositol synthase (dPIS) in a *CdsA* mutant background. The results of lipidomic and genetic analyses of the *CdsA* mutant highlight the importance of correct lipid composition during sperm development and show that phosphatidic acid levels are crucial in late stages of spermatogenesis.

## 1. Introduction

**Figure 1.**
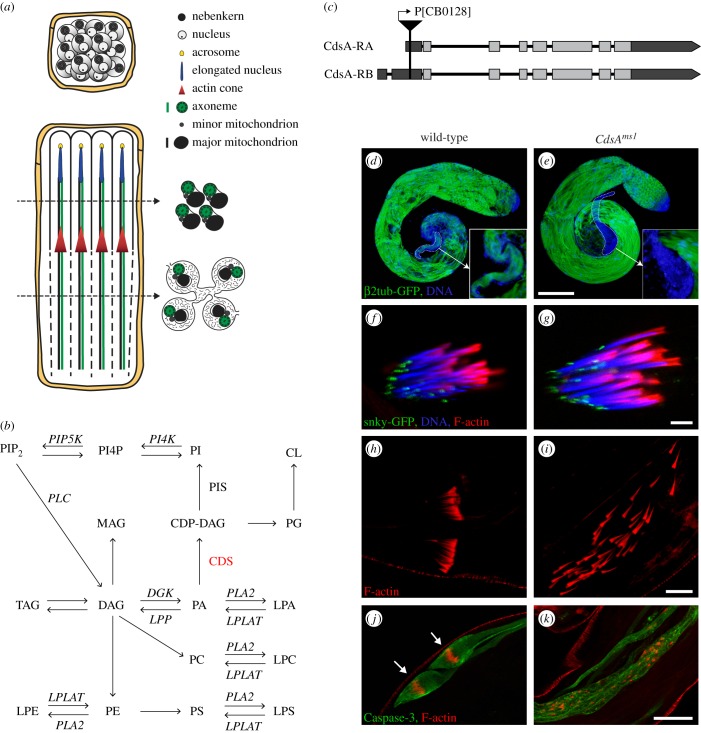
*CdsA^ms1^* mutant shows individualization defects. (*a*) Schematic of post-meiotic cysts before elongation and during individualization. Only four cells of the 64-cell cyst are shown during individualization. (*b*) Schematic of phospholipid biosynthesis. PA is a central component in the pathway. PA can be converted to DAG by Diacyl glycerol kinase (DGK) or to CDP-DAG by CDP-DAG synthase (CDS). CDP-DAG is the precursor for the synthesis of phosphatidylinositol (PI) by Phosphatidylinositol synthase (PIS) and phosphoinositides: PI4P and PIP_2_. CDP-DAG is also a substrate for phosphatidylglycerol (PG) and hence cardiolipin (CL) synthesis. DAG is the direct precursor of MAG, TAG, PC and PE. PLA_2_ and lysophospholipid acyltransferases (LPLAT) catalyse the production of lysophospholipids: LPA, LPC, LPS and LPE. (*c*) Schematic of *CdsA-RA* and *CdsA-RB* transcripts and the localization of the P[CB0128]-element in the *CdsA* gene. (*d–k*) Confocal images of wild-type (*d*,*f*,*h*,*j*) and *CdsA^ms1^* mutant (*e*,*g*,*i*,*k*) testes. (*d*,*e*) Microtubules in spermatid tails marked by *β*2-tubulin-GFP (green) are elongated in wild-type (*d*) and in *CdsA^ms1^* (*e*) testes, but mature sperm are missing from the seminal vesicle of the mutant (*d*,*e*, dashed line and inset). DNA stained with DAPI (blue). Scale bar, 100 µm. (*f*,*g*) Acrosome formation marked by snky-GFP (green), nuclear elongation stained by DAPI (blue) and investment cone assembly marked by phalloidin (red) are normal in both wild-type (*f*) and *CdsA^ms1^* (*g*) cysts. Scale bar, 10 µm. (*h*,*i*) Movements of investment cones marked by phalloidin (red) are synchronized in wild-type cysts (*h*), but disorganized in *CdsA^ms1^* mutant (*i*). Scale bar, 20 µm. (*j*,*k*) Cleaved Caspase-3 activity (green) is concentrated in the cystic bulge (arrows) of wild-type spermatids (*j*), but extended along scattered investment cones in the *CdsA^ms1^* cysts (*k*). Scale bars, 50 µm.

Many developmental processes rely on controlled membrane growth, but the precise regulation of this process has yet to be characterized. *Drosophila melanogaster* spermatogenesis is an ideal system to study this problem, because sperm development involves many different steps of membrane synthesis and remodelling. The primary spermatogonia divide four times and produce cysts with 16 connected cells. The meiotic divisions then produce a cyst with 64 round spermatids, where cells are still connected by intercellular cytoplasmic bridges [1,2]. Mitochondria are very important energy-supplying organelles for the mature sperm, whose morphogenesis during spermatogenesis is complex. The nebenkern forms after meiosis by the aggregation, fusion and wrapping of mitochondria, then divides into two parts (figure 1*a*) [3]. Both mitochondrial derivatives elongate together with the microtubule array that is formed around the mitochondrial surface [4]. Mature elongated sperm develop from approximately 12 µm spermatids through a process of intensive morphological changes. The spermatids increase their length 150-fold, producing spermatids approximately 1.8 mm long after elongation. The total surface area also increases fivefold during elongation and individualization of the spermatids [1,2,4]. Elongation from round to elongated spermatids includes extensive membrane biosynthesis, remodelling and regulated vesicular transport [3,5]. The 64 spermatids from each spermatogonium become polarized, and each spermatid has the nucleus at its apical end (figure 1*a*). Finally, the fully elongated spermatids undergo a synchronized individualization, in which each sperm within the cyst is covered by its own plasma membrane (figure 1*a*) [6]. This is assisted by the organization and movement of the investment cones, resulting in the formation of the cystic bulge that contains organelles, membranes and cytoplasm [7]. Following caspase activation in the cystic bulge, there is a degradation of cytoplasmic material within the spermatids [8]. Despite substantial progress in identifying factors involved in individualization, the nature of signals regulating its initiation and progression are still unknown. The large number of mutants with post-meiotic phenotypes implies that the final steps of spermatogenesis are especially sensitive to alterations in gene products [9].

Lipid composition and lipid–protein interactions are important factors affecting membrane curvature and function [10]. The lipid constituents of the cell membrane play either structural roles, such as phosphatidylcholine (PC) and cholesterol, or they can have both structural and signalling roles, such as phosphatidylethanolamine (PE) and phosphatidylserine (PS). The levels of lipids with a primarily signalling function, such as diacylglycerol (DAG), its phosphorylated derivative phosphatidic acid (PA) and phosphoinositides (PIP*_n_*), change in a highly regulated manner. PA and DAG facilitate both fusion and fission of cytoplasmic membrane compartments in endocytosis [11]. They function during exocytosis of membrane vesicles at the plasma membrane [12], in the budding of vesicles from the Golgi [13], and the fusion of mitochondrial membranes [14]. PIP*_n_* control several cellular events, such as cell proliferation, actin organization, membrane trafficking, cell differentiation and cell migration [15], but they also regulate lipid distribution [16]. The fact that the levels of PIP*_n_* are critical for spermatocyte cytokinesis is shown by the *fwd* (PI4 K*β*) and *giotto* (PI transporter) mutants, in which actin dynamics are disturbed during cytokinesis [17–20]. PIP_2_ is also known to be required for the elongation of spermatids, for basal body docking to the nuclear envelope and for axoneme formation [21,22]. Since lipid metabolic enzymes may function in many organs and developmental processes, mutations in them are expected to produce pleiotropic effects. Clearly, lipid metabolism is essential for normal sperm development, but the many roles that different lipids play are unclarified.

In eukaryotic cells, the endoplasmic reticulum (ER) produces most of the structural lipids [23]. From the ER, lipids are rapidly transported to other organelles, where they can be converted to organelle-specific lipids. The lipid composition of different organelle membranes varies significantly throughout the cell [24]. CDP-diacylglycerol synthase (CDS) is a very important enzyme in lipid biosynthesis that catalyses the synthesis of cytidine–diphosphate–diacyglycerol (CDP-DAG) from PA (figure 1*b*). Both the substrate and the product of the CDS enzyme are key branching points in the lipid biosynthetic pathway. *Drosophila* contains a single copy of CDS, encoded by the *CdsA* gene (CG7962), while vertebrates have two homologues, CDS1 and CDS2, both localized to the ER membrane [25]. *CdsA* was previously described to function in rhabdomere biogenesis in the *Drosophila* eye, based on the analysis of the hypomorphic *CdsA^1^* allele [26,27]. The *CdsA^1^* salivary gland exhibits an ectopic lipid droplet phenotype, and CdsA was recently proposed to be a coordinator of cell growth and lipid storage [28,29].

In this study, we show that a hypomorphic allele of *Drosophila CdsA* is male sterile with an individualization phenotype. Through lipidomic studies of the testis, we identify, in detail, the changes in lipid composition caused by the hypomorphic *CdsA* mutation. Our results reveal the significance of correct lipid composition during sperm individualization, a time when active membrane reorganization is occurring. It also highlights the importance of small signalling lipids, such as PA.

## 2. Results

### 2.1. Identification of male sterile alleles of the *Drosophila CdsA* gene

Upon screening of a P-element collection for new mutations in spermatogenesis, we found new, male sterile alleles of the *CdsA* gene. Homozygous male adults of the independently isolated *CdsA^CB-0128-3^* (called *CdsA^ms1^*) (figure 1*g*), *CdsA^EY08412^* and *CdsA^UM-8246-3^* alleles all show 100% sterility (electronic supplementary material, figure S1*a*), while females are fertile. All three alleles contain a P-element insertion in the 5′ untranslated region of the *CdsA* gene (electronic supplementary material, figure S2*a*) and show similar male sterile phenotype in homozygotes (electronic supplementary material, figure S1*a–d*), in the transheterozygous combination for each allele and for an overlapping deficiency (Df(3 L)BSC795), suggesting they are in the same complementation group. However, the fertile hypomorphic *CdsA^1^* allele [26] remained male fertile in the transheterozygous combination with the three male sterile alleles (*CdsA^CB-0128-3^*, *CdsA^EY08412^* and *CdsA^UM-8246-3^*), but showed male sterility over the Df(3 L)BSC795 deficiency. Thus, CdsA function seems to be very sensitive to gene dosage as a reduced level of *CdsA* expression leads to sterility, while complete loss of the protein in the null mutant *CdsA^GS8005^* causes early embryonic lethality [29]. There are two transcripts from the *CdsA* gene (*CdsA-RA* and *CdsA-RB*), and both transcripts are affected by the three P-element insertions (figure 1*c*). We tested the expression of the two transcripts using quantitative PCR in testes from wild-type and *CdsA^ms1^* mutants, and observed approximately 50% decrease in the *CdsA-RB* and *CdsA-RA-RB* mRNA levels (electronic supplementary material, figure S2*a*,*b*), indicating that *CdsA^ms1^* is a hypomorphic allele. The whole *CdsA-RA* sequence is included in the *CdsA-RB* sequence, therefore we cannot measure *CdsA-RA* alone.

The male sterile phenotype of the *CdsA^ms1^* allele is reversed completely by precise excision (*CdsA^ms1^* revertant) of the P element (electronic supplementary material, figures S1*e*,*f* and S2*c*). To rescue the male sterile phenotype, we generated a *P{UASp-CdsA-GFP}* transgenic construct where the coding region of *CdsA* was inserted into a fly transformation vector. Expression of the *P{UASp-CdsA-GFP}* transgene using the germline-specific *bam*-Gal4 driver rescued the male sterility on a *CdsA^ms1^* homozygous mutant background (electronic supplementary material, figures S1*g*,*h* and S2*c*), verifying that mutation in *CdsA* was responsible for the phenotype, as was the germline-specific reduction of CdsA.

We tested the subcellular localization of CdsA-GFP and found it to be enriched in ER (electronic supplementary material, figure S2*d*). This is consistent with the localization of mammalian CDS1 and CDS2 [25]. Membrane localization is to be expected because there are seven conserved transmembrane helices in the CdsA protein.

### 2.2. *CdsA* is required for proper spermatid individualization

To identify the cause of sterility in homozygous *CdsA^ms1^* males, we studied each stage of spermatogenesis. In the mutant testis, as in wild-type, proper cell divisions result in a cyst with 64 synchronized spermatocytes that then start to elongate. Using a *β*2-tubulin-EGFP transgenic line to fluorescently label microtubules, we observed that the elongation of axonemes was completed, but the maturation process did not complete and no fully formed sperm were found in the seminal vesicles of *CdsA^ms1^* mutants (figure 1*d*,*e* and inset). Acrosomes formed normally at the tip of each elongated nucleus, both in the control and in the *CdsA^ms1^* mutant (figure 1*f*,*g*).

To determine if CdsA is required for individualization, we visualized the investment cones that mainly consist of actin [6]. In the wild-type testis, these cones assemble around the spermatid nuclear bundle (figure 1*f*) and move synchronously to the basal end of the elongated cyst (figure 1*h*), during which time every spermatid becomes surrounded by its own plasma membrane. In the testis of homozygous *CdsA^ms1^* males actin-rich cones are established behind the 64 elongated nuclei (figure 1*g*), but they move asynchronously along the elongated spermatids (figure 1*i*; electronic supplementary material, figure S2*c*). Similarly, we observed dispersed actin cones in the testes of the other two alleles of *CdsA*, *CdsA^EY08412^* and *CdsA^UM-8246-3^* (electronic supplementary material, figure S1*a*–*d*). The Lasp protein is required to tether the actin cytoskeleton to the plasma membrane around the investment cones; it co-localizes with filamentous actin [30]. We found that Lasp was correctly localized in *CdsA^ms1^* testes (electronic supplementary material, figure S3*a*), indicating that investment cone formation and its tethering to the actin cytoskeleton were normal. During spermatid individualization most of the cytoplasm and cellular organelles in the cystic bulge are broken down by a Caspase-3-mediated non-apoptotic degradation pathway and deposited into waste bags [8]. In order to visualize the cystic bulge and the individualization complex, we stained testes with an antibody specific to the active form of Caspase-3. In *CdsA^ms1^* mutants, Caspase-3 enzyme is activated extensively along the elongated spermatids around the dispersed investment cones, but neither regular cystic bulges nor proper waste bags were seen (figure 1*j*,*k*; electronic supplementary material, figure S2*c*). We tested the individualization phenotype in testes from *P{UASp-CdsA-GFP}*;*bam*-Gal4, *CdsA^ms1^* homozygous males and found that rescue of male sterility correlates with rescue of individualization (electronic supplementary material, figures S1*g*,*h* and S2*c*).

### 2.3. *CdsA^ms1^* mutant shows abnormal mitochondria in elongated spermatids

**Figure 2.**
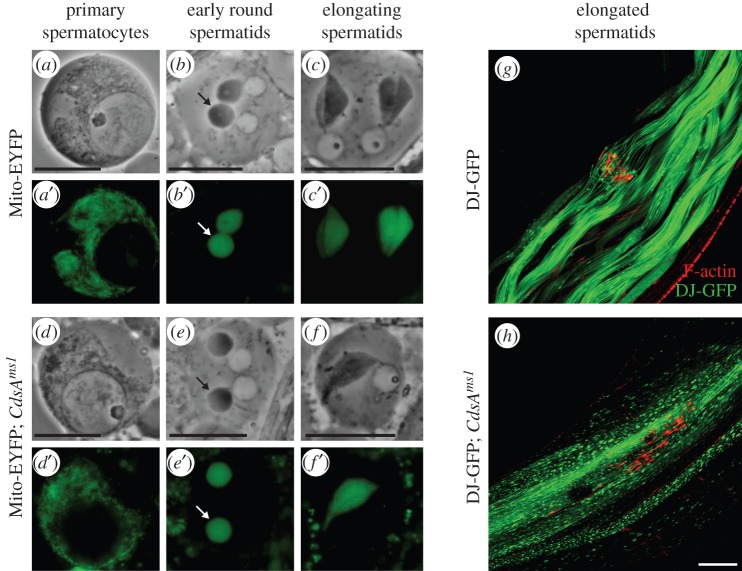
Mitochondria of *CdsA^ms1^* mutants are affected in elongated spermatids. (*a*–*f*) Phase-contrast (*a*–*c*,*d*–*f*) and corresponding fluorescent images (*a*′–*c*′,*d*′–*f*′) of live squashed preparations of spermatocytes (*a*,*a*′,*d*,*d*′) and spermatids (*b*,*b*′,*c*,*c*′,*e*,*e*′,*f*,*f*′) expressing Mito-EYFP (green) in wild-type (*a–c*) and in *CdsA^ms1^* (*d–f*) mutant showing normal morphology in mitochondrial fusion, nebenkern formation (*b*,*e*, arrows) and elongation (*c*,*c*′,*f*,*f*′). Scale bar, 10 µm. (*g–h*) Confocal micrographs of elongated spermatid cysts expressing DJ-GFP (green) and stained for F-actin (red). In wild-type (*g*), investment cones are synchronized and mitochondrial DJ-GFP distribution is smooth. In *CdsA^ms1^* mutants (*h*), investment cones are dispersed and DJ-GFP distribution resembles pearl-like structures in mitochondria. Scale bar, 40 µm.

To uncover the cellular effects of the *CdsA^ms1^* mutation, we tested the morphological changes of mitochondria, one of the centrally important organelles of mature sperm. In order to follow the morphological changes of mitochondria during spermatogenesis, we used two transgenic reporters: Mito-EYFP in early mitotic and meiotic stages, and GFP-tagged Don Juan protein (DJ-GFP) in the elongated spermatids [31,32]. In the testis of *CdsA^ms1^* mutants, we did not detect any changes in the primary spermatocytes (figure 2*a*,*d*) in nebenkern formation (figure 2*b*,*e*), nor in the elongation of the two mitochondrial derivatives in post-meiotic spermatids (figure 2*c*,*f*) when compared with the wild-type.

**Figure 3.**
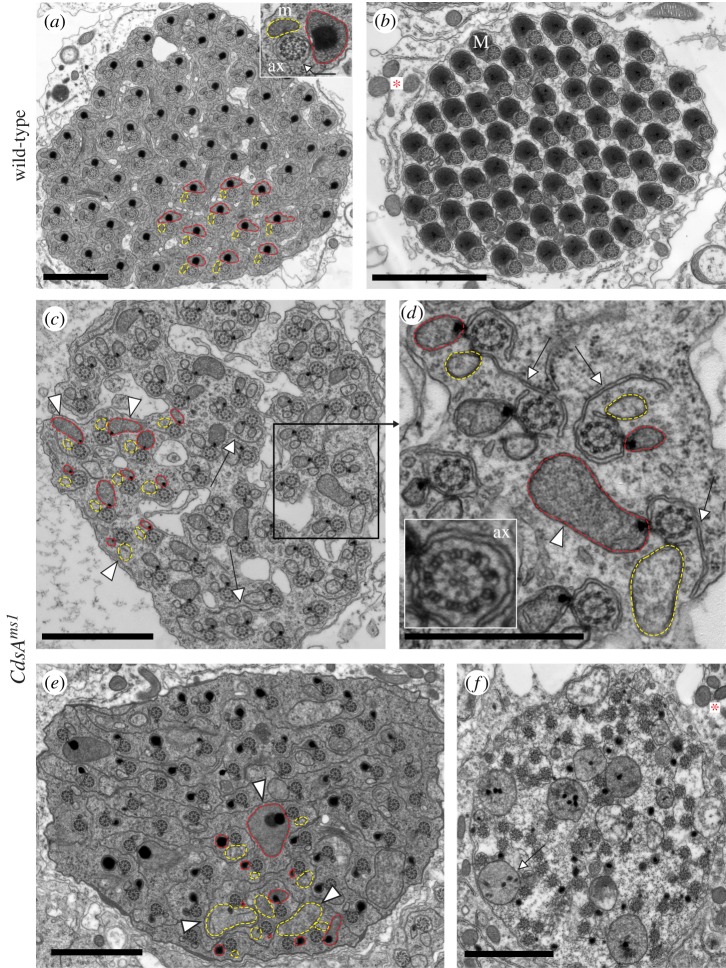
Ultrastructure of spermatids shows morphological changes in *CdsA^ms1^* testes. (*a–f*) Transmission electron micrographs of cross-sections of elongated spermatid cysts in wild-type (*a*,*b*) and in *CdsA^ms1^* (*c–f*) testes. (*a*,*b*) Elongated cyst contains 64 synchronized spermatids. Axonemal sheath is marked by arrow. Axonemes (ax), major (M) (in red) and minor (m) (in yellow) mitochondrial derivatives before (*a*) and after (*b*) individualization. Mitochondria of somatic cells are marked with an asterisk. (*c*–*e*) Representative cross-sections of elongated pre-individualized *CdsA^ms1^* cysts contain 64 unsynchronized spermatids either with same-sized mitochondrial derivatives or with enlarged, different-sized mitochondrial derivatives (arrowheads) and overgrowth of the axonemal sheath (arrows). (*d*) Inset of (*c*), the structure of the axoneme (ax) remained intact in the *CdsA^ms1^* mutant (inset) with the overgrowth of the axonemal sheath (arrows). (*f*) *CdsA^ms1^* cysts show disorganized cytoplasm with overgrowth of mitochondria (arrow). (*b*,*f*) Somatic mitochondria show normal structure (asterisk). Scale bars, 2 µm in (*a*–*c*,*e*,*f*) and 1 µm in (*d*).

The distribution of DJ-GFP throughout individualization is evenly dispersed in the wild-type testis (figure 2*g*). In contrast, we always observed a pearl-like structure in *CdsA^ms1^* testes, which only occurs in elongated, but not in mature spermatids in wild-type (figure 2*h*). Analysis of the structural changes of elongated pre-individualized spermatogenic cysts in the *CdsA^ms1^* mutant using electron microscopy also revealed abnormalities (figure 3*c*–*f*). The 64 intact axonemes can be seen to have normal microtubule arrangement in the cross-section of *CdsA^ms1^* mutant testes (figure 3*a*–*e*). After individualization, wild-type spermatids are closely apposed, having their own plasma membrane, and the major mitochondrial derivatives are filled with paracrystalline (figure 3*b*). However, both the major and minor mitochondrial derivatives associated with the axonemes show overgrowth phenotypes in *CdsA^ms1^* mutant testes. One axoneme is often associated with two mitochondrial derivatives of similar size, or one of these becomes extremely large with or without paracrystalline formation (figure 3*c*,*d*, arrowheads). The size of mitochondrial derivatives is not uniform, and an overgrowth of endomembranes, such as the axonemal sheath (figure 3*d*, arrows), is also a typical phenotype observed in the mutant. The grossly disorganized *CdsA^ms1^* mutant cysts fail to undergo proper individualization, and they have excess cytoplasm and lack their own plasma membranes (figure 3*e*,*f*). We found a large amount of cytoplasm in which many axonemes and abnormally sized mitochondria are immersed (figure 3*f*, arrow), most probably leading to cell death. To confirm this, we used terminal deoxynucleotidyl transferase-mediated dUTP nick-end labelling (TUNEL) and found abundant TUNEL positive labelling in the elongated cysts of the *CdsA^ms1^* mutant (electronic supplementary material, figure S3*b*). Defect in sphingolipid metabolism also resulted in cell death and increased TUNEL positive structures in *Drosophila* testis [33].

Interestingly, in *CdsA^ms1^* mutants, the somatic cells of the testis contain normal mitochondria with respect to both their size and structure (figure 3*b*,*f*, asterisk). To test the function of somatic mitochondria, we performed flight and climbing assays, and found no differences between *CdsA^ms1^* mutant and wild-type flies (98% of *CdsA^ms1^* mutants fly normally) (electronic supplementary material, figure S3*c*).

### 2.4. The hypomorphic *CdsA^ms1^* allele perturbs lipid biosynthesis

**Figure 4.**
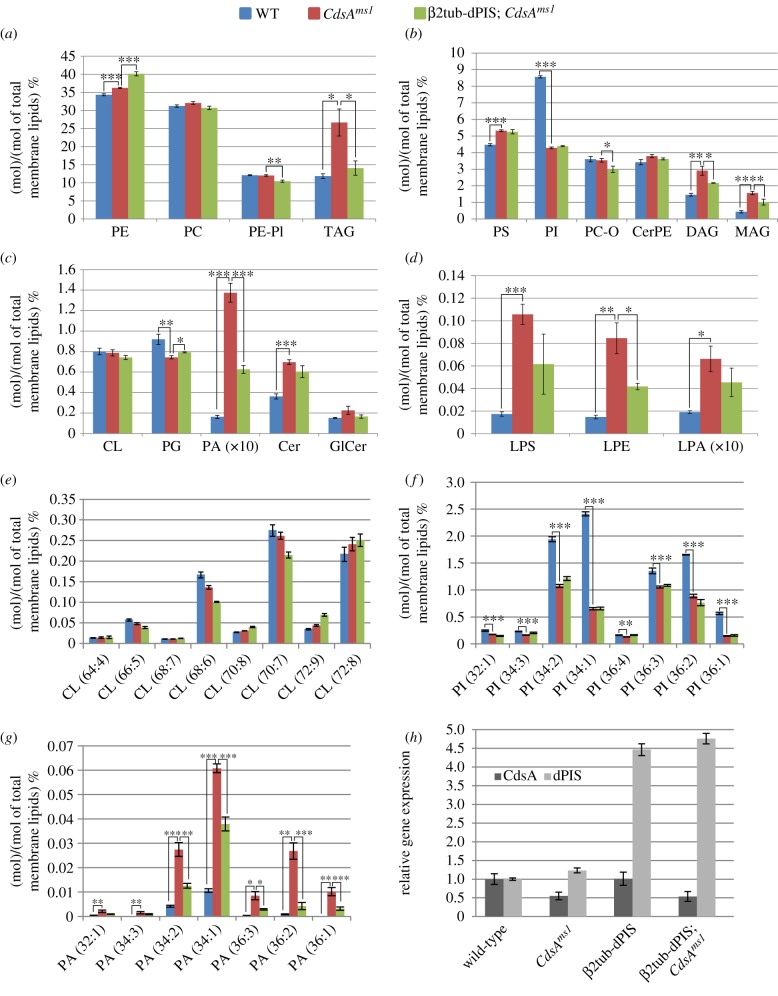
Changes in lipid classes and lipid molecular species in *CdsA^ms1^* and *β*2tub-dPIS; *CdsA^ms1^* testes. (*a*–*g*) Lipid class and species changes in wild-type (WT), *CdsA^ms1^* mutant and *β*2tub-dPIS; *CdsA^ms1^* mutant testes by mass spectrometry. (*a*) Lipid class distribution of the main lipid classes: (*a*) PE, PC, PE-PI, TAG, (*b*) PS, PI, PC-O, CerPE, DAG, MAG, (*c*) minor lipid classes: CL, PG, PA, Cer, GlCer and (*d*) lysophospholipid classes: LPS, LPE, LPA. Lipid species distribution of (*e*) CL, (*f*) PI, (*g*) PA. The levels are normalized to total membrane lipids. Error bars indicate mean ± s.e.m., *n* = 5. *p < 0.05; **p < 0.01; ***p < 0.001 (Student's *t*-test). (*h*) Relative expression levels of *CdsA* and *dPIS* transcripts were quantified by quantitative RT-PCR in the testis of wild-type, *CdsA^ms1^* homozygotes, β2tub-dPIS and β2tub-dPIS; *CdsA^ms1^*.

Mass spectrometry-based lipidomic analysis allows one to follow tissue-specific changes in the *Drosophila* lipidome [33,34,35]. To assess the alterations that may underlie the phenotypic differences between the wild-type and *CdsA^ms1^* mutant *Drosophila* testes, we applied high-sensitivity, high-resolution mass spectrometric shotgun lipid profiling. We identified and quantified 166 lipid species from 18 lipid classes (figure 4*a*–*d*; electronic supplementary material, table S1).

As expected, the testis lipidome is significantly altered in comparison with the wild-type (figure 4*a*–*d*; electronic supplementary material, table S1). The product of CdsA, CDP-DAG, is split between PI and PG synthesis; the latter leads to the formation of CL (figure 1*b*). Upon reduction of CdsA activity, PG showed a small, but statistically significant decrease at the lipid class level (figure 4*c*). Due to the obvious morphological changes in the mutant mitochondria, we also expected large changes in the levels of the mitochondrial lipid CL. This lipid is involved in the biogenesis, dynamics and supramolecular organization of mitochondrial membranes, and in their bioenergetic processes [36]. There was no change in the overall amount of CL or in the levels of CL lipid species (figure 4*c*,*e*).

**Figure 5.**
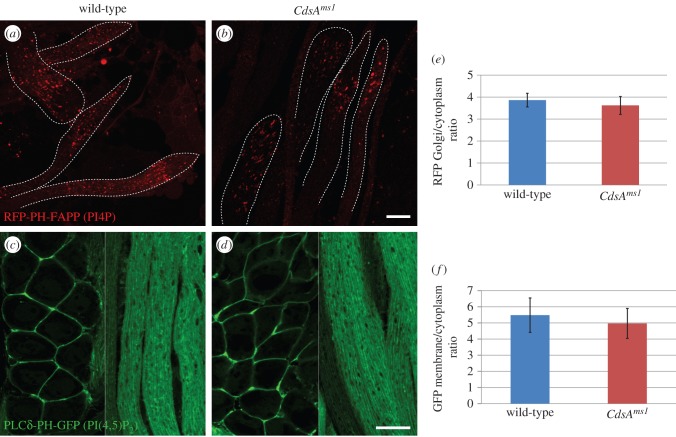
Distribution of phosphoinositides in *CdsA^ms1^* male germ cells. (*a*,*b*) Confocal micrographs of the squashed and fixed preparations of elongated spermatids expressing the PI4P-binding RFP-PH-FAPP transgene. RFP-PH-FAPP localizes to punctate structures both in the wild-type (*a*) and *CdsA^ms1^* (*b*) elongated spermatids. (*c–f*) Confocal micrographs of male germ cells expressing PLC*δ*-PH-GFP, which binds PI(4,5)P_2_. In spermatocytes (left side of images) and in elongated spermatids (right side of images), PI(4,5)P_2_ is localized to the plasma membrane in wild-type (*c*) and *CdsA^ms1^* (*d*) testes. Images were taken with the same exposure time in the case of each transgene, with or without the *CdsA^ms1^* homozygotes. Membrane to cytoplasmic RFP intensity (*e*) or GFP intensity (*f*) ratios were calculated from measurements of mean pixel intensities within the same sized area of membrane versus cytoplasm. Scale bars, 20 µm.

One of the biggest changes occurred in the level of PI (figure 4*b*). It showed a highly significant and species-dependent reduction, with PI (34 : 1) being both the most abundant and the most affected species (figure 4*f*). PI is phosphorylated into PIP*_n_* that are specifically enriched in different compartments (plasma membrane, Golgi, early and late endosomes) and can be used as markers for these organelles. Importantly, PI(4,5)P_2_ is a substrate of phospholipase C (PLC), resulting in the production of DAG, a portion of which is used for PA synthesis (figure 1*b*). Although the applied shotgun lipidomic approach is not suitable to quantify PIP*_n_*, we were able to analyse their localization by using *in vivo* fluorescent markers for two types of PIP*_n_*, PI4P and PI(4,5)P_2_, in a *CdsA^ms1^* homozygous mutant background [18,37]. The punctate distribution pattern of the PI4P-binding RFP-PH-FAPP marker protein was preserved in the mutant, and the Golgi to cytosolic RFP intensity ratio was also similar in wild-type and *CdsA^ms1^* spermatids (figure 5*a*,*b*,*e*). However, in testes from *CdsA^ms1^* homozygotes, the overall RFP intensity is lower for unknown reasons. Similarly, we have not observed any obvious differences in the plasma membrane localization of the PI(4,5)P_2_ specific PLC*δ*-PH-GFP in wild-type and *CdsA^ms1^* testes (figure 5*c*,*d*,*f*).

Reduction in CdsA activity causes a sevenfold increase of total PA at the overall lipid class level in *CdsA^ms1^* testes (figure 4*c*), which happens in a strongly species-dependent manner, with PA(34 : 1) being the most abundant and PA(36 : 1) being the most responsive species (figure 4*g*). The elevation of PA seems to affect the lipogenic pathway and results in increased DAG levels (more than twofold) (figure 4*b*). Similarly to PA, DAG also lies at a key branching point in lipid metabolism. Indeed, all the lipids in direct biosynthetic connection with DAG were elevated, such as TAG (twofold), MAG (more than threefold) and slightly, but significantly, PE as well. Moreover, PS, which is synthesized from PE, also increased (figure 4*a*,*b*). In addition, very interesting alterations were detected in different lysophospholipids, products of phospholipase A (PLA) enzymes, especially LPE and LPS. The levels of these minor lipids were elevated about sixfold. The alteration of LPA levels, although smaller in extent, is worth mentioning (figure 4*d*). Concerning sphingolipids, Cer was significantly increased (figure 4*c*), which could be a result of the increased cell death observed by TEM and TUNEL staining in the *CdsA^ms1^* testes.

We measured the lipid composition of the revertant line of *CdsA^ms1^* and found that the rescue of the male sterile phenotype is associated with the restoration of the lipid profile close to the WT pattern (electronic supplementary material, figure S3*d*).

### 2.5. Phosphatidic acid level is critical for the late stages of spermatogenesis

Because all PI and PA species are affected in *CdsA^ms1^* testes (figure 4*f*,*g*), we hypothesized that these particular changes could cause the male sterile phenotype. *Drosophila* PI synthase (dPIS) produces PI from CDP-DAG (figure 1*b*), and its overexpression partially suppresses the retinal degeneration phenotype in the eye of the *cds^1^* mutant [38]. To test our hypothesis, we overexpressed dPIS in a *CdsA^ms1^* mutant background. We generated a *β*2tub-dPIS construct and established transgenic lines. Gene expression from the *β*2tub promoter was entirely restricted to germ cells and was increased four- to fivefold when compared with endogenous gene expression (as measured by quantitative RT-PCR; figure 4*h*). Importantly, we found that *β*2tub-dPIS overexpression suppresses the male sterile phenotype of *CdsA^ms1^* mutants in 65% of the males (*n* = 192) (electronic supplementary material, figures S1*i*,*j* and S2*c*).

Lipidomic analysis was applied again to see which lipid changes might be responsible for the suppression of male sterility. Surprisingly, we did not find any significant changes in PI at lipid class nor at the molecular species level in the *β*2tub-dPIS; *CdsA^ms1^* testes (figure 4*b*,*f*). However, there was a substantial change in PA. In comparison with its dramatically elevated level in *CdsA^ms1^* testis, the total PA level was halved in *β*2tub-dPIS; *CdsA^ms1^* mutant males (figure 4*b*). Restoration of normal PA levels was species-dependent: the detected loss was 40% in the main species PA(34 : 1), and more than 80% in PA(36 : 2) (figure 4*f*).

Therefore, the testis-specific elevation of PA levels could be responsible for the male sterile phenotype in *CdsA^ms1^* mutants. Further lipid changes that could account for the rescue were detected in lysophospholipids, and in all neutral lipids (figure 4*b*–*d*; electronic supplementary material, table S1). All these components showed opposing alterations upon dPIS overexpression as compared with those observed in the *CdsA^ms1^* mutants. To rule out the possibility that the overexpression of dPIS somehow upregulated *CdsA* itself, we measured *CdsA* transcript levels by quantitative RT-PCR and found no significant difference in the expression level of *CdsA* mRNA in *β*2tub-dPIS; *CdsA^ms1^* compared with *CdsA^ms1^* testes (figure 4*h*). We found a small, but reproducible increase in the dPIS mRNA level in *CdsA^ms1^* testes (figure 4*h*). Taken together, these data suggest that CdsA is crucial for the fine-tuning of lipid composition during spermatogenesis, and it acts by controlling the amounts of signalling lipids.

## 3. Discussion

The testis is a lipid-rich organ, and spermatids undergo extensive membrane reorganization during differentiation. Here, we focused on a key enzyme in lipid metabolism, CDP-DAG synthase, and investigated its importance in spermatogenesis. The hypomorphic *CdsA^ms1^* allele shows an individualization phenotype in the germ line, while somatic tissues are unaffected. It is known that elongation and individualization of spermatids take place after meiosis when only a low level of transcription is observed, and only a few loci are activated [39,40]. Therefore most of the gene products necessary for the post-meiotic stages are synthetized earlier. The transcriptional shut-off in post-meiotic stages, during spermatid elongation and individualization, makes these late stages of spermatogenesis particularly sensitive to even a small reduction in gene products (in somatic cells such a reduction is without effect). Although *CdsA* function is not required for individualization complex formation, it may play an indirect role in its movement. Mitochondrial asynchrony and overgrowth, as well as the enlargement of endomembranes of spermatids, are the most prominent aberrations seen in the *CdsA^ms1^* testes. It seems possible that the large size of mitochondrial derivatives in *CdsA^ms1^* mutants causes steric hindrance that prevents the passage of investment cones. Mutations in genes, such as *parkin*, which cause a different type of abnormality in the mitochondria, also lead to defective progression of the individualization complex and to a failure of sperm maturation [41]. Our testis-specific lipidomic analysis showed reduced PI and elevated PA level in *CdsA^ms1^* mutant testis. Although we measured a strong decrease in PI levels, we did not find changes in the localizations of PIP*_n_*, such as PI4P and PI(4,5)P_2_. PA is a low-abundance constituent of all living organisms. *Drosophila* tissues show remarkable tissue-specificity for PA. It is almost undetectable in fat body and brain, but gut and wing discs contain substantial amounts [33]. *CdsA^ms1^* mutant testis shows dramatic elevation in PA both at the lipid class and the lipid species level. As a cone-shaped structure, PA can generate negative curvature in membranes, and participates in fusion and fission events [42]. The fusogenic role of PA in mitochondria was established in mammalian cells by characterizing the mitochondrial PLD protein [14]. CdsA is localized in the ER as an integral membrane protein. The contact sites between the ER and mitochondria are a highly conserved feature of eukaryotic cells and have been associated with several important functions, including mitochondrial division [43]. Prior to spermatid individualization, the axoneme is surrounded by a cylindrical sheath of ER, called the axonemal sheath, whose outer membrane is in contact with the mitochondrial derivatives along its entire length [3]. We propose that PA could be enriched at the axonemal sheath–mitochondria contact sites and induce the overgrowth of the ER and mitochondrial membranes in *CdsA^ms1^* testes. One possible mechanism of action whereby PA regulates mitochondrial dynamics was proposed in a study that tested *in vivo* function of mouse PLA_1_ enzyme [44]. Knocking out the *PA-PLA_1_* gene caused defects in the organization of mitochondria during spermiogenesis, leading to sperm malformation and male subfertility. This is in agreement with our findings and suggests that the level of PA should be kept low to ensure proper mitochondrial function in spermatogenesis.

The loss of CdsA function was investigated in different *Drosophila* tissues. It was shown that the elevation of a single PA species (34 : 2 PA) is able to disrupt membrane transport to the apical domain in *Drosophila* photoreceptors and cause defective rhabdomere biogenesis in *cds^1^* hypomorphic mutants, without an increase in total retinal PA levels [27]. In another study, *CdsA* RNAi led to doubled PA levels in whole larvae, whereas no change was seen in the salivary gland where CdsA regulates fat storage and cell size [29]. It should be noted here that such ‘discrepancies’ between results from different tissues may represent different tissue-specificity and/or activity of lipid metabolic enzymes that act on the same substrate.

Besides the obvious involvement of PA, it should be emphasized that many other alterations happened to lipid species and/or lipid class levels in the *CdsA^ms1^* mutant, and these could also serve to disrupt the system. Such changes occurred in PG, LPE, LPS and LPA, and also in MAG and DAG. Similarly to PA, lysophospholipids are not simple metabolic intermediates, but exhibit biological properties resembling those of extracellular growth factors or signalling molecules [45]. Lysophospholipids are post-synthetically modified in a process known as the Lands cycle that involves hydrolysis and re-acylation/transacylation steps. A double mutant of two reacylation enzymes, *oys;nes*, shows pleiotropic phenotypes, including a spermatid individualization phenotype but with normal mitochondrial and axonemal sheath morphology [46]. However, the early movement of investment cones was affected in the *oys;nes* double mutant, suggesting that the investment cone progression is very sensitive to alterations in the amounts of lysophospholipids. Overexpression of PI synthase in *CdsA^ms1^* testes suppresses male sterility. Our experiments show that PI synthase somehow, probably indirectly, induces the reduction of PA without the upregulation of *CdsA* transcription itself. In a human cell line, the overexpression of the *pis1* gene led to the overproduction of both PI synthase and PI : inositol exchange reactions, indicating that the Pis1 enzyme catalyses both of these activities. However, it neither enhanced the rate of PI biosynthesis, nor resulted in a significant increase in the cellular levels of PI [47]. This is consistent with our results showing that overexpression of dPIS did not elevate the overall amount of PI on a *CdsA^ms1^* background. It is possible that there exists another protein with CDP-DAG synthase activity, which could use PA as a substrate. Indeed, recently, Tam41, a highly conserved mitochondrial maintenance protein, was identified in yeast, which directly catalyses the formation of CDP-DAG from PA in the mitochondrial inner membrane [48]. We tested the expression of the potential *Drosophila* orthologue of the *Tam41* gene, *CG33331*, and did not find elevated transcript levels in *β*2tub-dPIS; *CdsA^ms1^* testes (B.L. 2015, unpublished data), but we cannot rule out the possibility of an increase in the level or activity of CG33331 protein.

The question of how dPIS overexpression could change the level of PA, via even a potential alternative pathway, is still unanswered. It was shown in yeast that the amount of PA can transcriptionally regulate the level of PI synthase. The soluble transcriptional repressor Opi1p is inhibited by binding to PA at the ER, but can rapidly translocate into the nucleus in response to inositol-induced PA consumption [49]. In the *Drosophila* retina, a mutation of the lipid phosphate phosphohydrolase led to elevated PA levels, which were coupled with PI synthase transcript upregulation [50]. This suggests that the level of PA and the level of PI synthase transcription are somehow connected with each other. This is consistent with our results, where dPIS is slightly upregulated in the *CdsA^ms1^* mutant. However, because of the transcriptional shut-off after meiosis, the cells are probably not able to compensate the elevated PA level in the mutant by increasing the amount of dPIS and other factors. One possible strategy to find the precise biochemical pathway responsible for the suppression of male sterility of *CdsA^ms1^* by overexpression of dPIS would be to correlate the lipidomic observations with testis-specific gene expression or proteomic analysis of transcription factors, as well as lipid metabolic enzymes and their regulators.

Our study demonstrates that a mutation in a single lipid biosynthetic gene may have pleiotropic effects on lipid metabolism, which together may lead to severe phenotypic alterations, such as male sterility. Lipidomic analysis revealed that the observed phenotypic changes in *Drosophila* testes can be caused by a comprehensive reorganization of the testis lipidome, and also suggests that the fruit fly testis is an ideal and sensitive system to study changes in lipid composition and their effects on development.

## 4. Material and methods

### 4.1. Fly stocks and mutants, transgenes, fertility test and locomotor assays

Flies were crossed and maintained on standard cornmeal agar medium at 25°C. The *w^1118^; P{RS3}CdsA^CB-0128-3^* (*CdsA^ms1^* allele), *w^1118^; P{RS3} CdsA^UM-8246-3^* male sterile alleles of *CdsA* (CG7962) were isolated from a P-element insertion collection (Szeged Stock Center). The *β*2-tubulin-GFP line was provided by David M. Glover. Lines expressing Snky-GFP, Plc*δ*-PH-GFP and RFP-PH-FAPP were previously described [18,37,51], and generously provided by Julie A. Brill. All other stocks were obtained from the Bloomington Stock Center: *w^1118^; Df(3 L)BSC795/TM6C, Sb^1^ cu^1^, y^1^ w^67c23^; P{EPgy2}CdsA^EY08412^*, w*; P{sqh-EYFP-Mito}3, w*; P{dj-GFP.S}AS1/CyO, and from the Kyoto Stock Center: *y^1^ w^67c23^; P{GSV3}CdsA^GS8005^/TM3, Sb^1^ Ser^1^*. Plc*δ*-PH-GFP, RFP-PH-FAPP and Mito-EYFP lines were recombined to the *CdsA^ms1^* allele on the third chromosome.

The C-terminal *P{UASp-CdsA-GFP}* construct was generated by the Gateway cloning system (Invitrogen) according to the manufacturer's protocol. The HL01284 cDNA clone (DGRC) was used for PCR amplification (electronic supplementary material, table S2) and cloned into the pPWG vector (DGRC). Transgenic lines were established and a *bam*-Gal4 testis-specific driver [52] was used (kindly provided by Helen White-Cooper) to express the transgene on the *CdsA^ms1^* mutant background. The *P{*β*2tub-dPIS}* transgenic construct (*β*2tub-dPIS) was generated by amplifying the *dPIS* cDNA (electronic supplementary material, table S2). The PCR fragment was cloned into testis-vector3 [37] (kindly provided by J. A. Brill) using *NotI* and *XbaI* restriction sites and subsequently verified by sequencing. Transgenic flies were generated by injection of *w^1118^* embryos, mapped and combined with the *CdsA^ms1^* allele.

Remobilization of the *P{RS3}CdsA^CB-0128-3^* element was done according to Engels *et al.* [53]. Revertant lines were tested for fertility and the precise excisions of the *P{RS3}CdsA^CB-0128-3^* were further confirmed by PCR. Oregon-R stock was used as the wild-type control and *w; P{*β*2tub-dPIS}/CyO; CdsA^ms1^/TM3* in the suppression experiment. We performed the fertility tests by crossing single males with four wild-type females. The progeny was counted in every tube and the average was calculated from 30 to 50 males (in the case of *P{*β*2tub-dPIS}* overexpression *n* = 192).

Flight tests were carried out with 3–5-day-old flies (*n* = 100/genotype) in a flight chamber and scored for the ability to fly up, horizontally or down, as described in [54]. For the climbing assay, 3–5-day-old flies (*n* = 100/genotype) were transferred to a clean measuring cylinder (five flies per measure), and the time they needed to climb up 10 cm length was recorded, then velocity was determined in cm s^−1^.

### 4.2. Staining and microscopy

For phase-contrast microscopy and for immunostaining, intact or partially squashed testes from 2 to 4 days old wild-type and mutant flies were processed as described earlier [55]. DAPI (1 µg ml^−1^) was used for DNA staining and Texas Red-X Phalloidin (Invitrogen) was used in 1 : 250 dilution for actin visualization. Primary antibodies used: rabbit anti-Lasp, 1 : 200 (gift from Anne Ephrussi); mouse anti-Calnexin99A, 1 : 100 (gift from Sean Munro); rabbit anti-cleaved-Caspase3, 1 : 200 (clone 5A1E, Cell Signalling). Alexa Fluor 488 conjugated anti-rabbit secondary antibody was from Invitrogen. TUNEL labelling of the testis was carried out using Click-iT TUNEL Alexa Fluor Imaging Assay (Invitrogen) as described in Kibanov *et al.* [56]. The samples were mounted in Fluoromount (Southern Biotech) and imaging was done with an Olympus BX51 fluorescent microscope or with an Olympus FV 1000 confocal microscope. Signal quantification for RFP-PH-FAPP and PLC*δ*-PH-GFP was done on images collected using the same exposure time for different genotypes. Membrane to cytoplasmic fluorescent signal ratios were calculated from the measurements of mean pixel intensities within equal areas of membrane versus cytoplasm (*n* = 20). Measurements were done using ImageJ software.

### 4.3. Quantitative RT-PCR

Total RNA was extracted from 25 pairs of testis per genotype using the RNeasy Mini Kit (Qiagen) according to the manufacturer's instructions. The whole RNA sample was transcribed into first strand cDNA using the RevertAid First Strand cDNA Synthesis Kit (Thermo Scientific) according to the manufacturer's instructions. For quantitative RT-PCR assays, 20 µl Luminaris Color HiGreen qPCR Master Mix (Thermo Scientific) was used with 0.3 µM of each primer (electronic supplementary material, table S2) and a CFX96 Real-Time PCR Detection System (Bio-Rad). The expression values were normalized to an internal control of *rp49* mRNA for each sample. The final values represent the mean (and standard error) of triplicates.

### 4.4. Electron microscopy

Testes were dissected and fixed overnight at 4°C in 3.2% paraformaldehyde, 1% glutaraldehyde, 1% sucrose, 0.028% CaCl_2_ in 0.1 N sodium cacodylate (pH = 7.4), thoroughly washed in 0.1 N sodium cacodylate (pH = 7.4), post-fixed in 0.5% osmium tetroxide for 1 h, and embedded in Durcupan (Fluka) resin according to the manufacturer's recommendations. Seventy-nanometre sections were cut from two to three testes per genotype, stained in Reynold's lead citrate and evaluated using a JEM-1011 electron microscope (JEOL) equipped with a Morada camera and iTEM software (Olympus).

### 4.5. Lipid analysis

Lipid standards were obtained from Avanti Polar Lipids (Alabaster, AL, USA). The solvents used for extraction and for mass spectrometric analyses were of liquid chromatographic grade from Merck (Darmstadt, Germany) and Optima LC-MS grade from Thermo Fisher Scientific (Waltham, MA, USA). All other chemicals were purchased from Sigma and were of the best available grade. Lipid species were annotated according to the updated comprehensive classification system for lipids [56].

For lipid extraction we used five pairs of testes per replicate. Five independent replicates were analysed in each genetic background. The samples were sonicated in 400 µl methanol (containing 0.001% butylated hydroxytoluene as antioxidant) for 5 min, shaken for another 5 min, and finally centrifuged at 13 000 r.p.m. for 5 min. The supernatant was transferred into a new Eppendorf tube and stored at −20°C until mass spectrometric analysis.

### 4.6. Mass spectrometry

Mass spectrometric analyses were performed on a LTQ-Orbitrap Elite instrument (Thermo Fisher Scientific, Bremen, Germany) equipped with a Dionex WPS-3000TPL RS temperature-controlled autosampler (Thermo Scientific) and a nano ESI source. Ionization voltages were +1.8 kV and −1.8 kV in positive and negative ion modes, respectively. The temperature of the ion transfer capillary was 300°C. Acquisitions were performed at the mass resolution *R_m_*_/*z*__400_ = 240 000.

For quantification, 20 µl of the lipid extract was spiked with an internal standard mix containing 114 pmol PC D31-16 : 0/18:1, 40 pmol PE D31-16 : 0/18 : 1, 17 pmol PI D31-16 : 0/18 : 1, 30 pmol PS D31-16 : 0/18 : 1, 4 pmol PG D31-16 : 0/18 : 1, 1 pmol PA D31-16 : 0/18 : 1, 2 pmol CL 56:0, 8 pmol Cer d18 : 1/12 : 0, 6 pmol CerPE d17 : 1/12:0, 8 pmol GluCer d18 : 1/12 : 0, 4 pmol MAG 19 : 1, 1 pmol DAG 44 : 2, 1 pmol TAG 51 : 3 before the MS measurement. The spiked extracts were diluted with 280 µl chloroform : methanol 1 : 3 (by vol.) containing either 5% dimethyl-sulfoxide (as an additive in the negative ion mode) or 7.5 mM ammonium chloride (as an additive in the positive ion mode). Three microlitres of sample was infused at a flow rate of 300 nl min^−1^, and data were acquired from 1.6 to 5.0 min. The following lipid classes were detected and quantified in the positive ion mode: PC (diacyl), PC-O (alkyl-acyl), LPC, hexosyl ceramide (HexCer), MAG, DAG and TAG. PE (diacyl), PE-Pl (plasmalogen PE-alkenyl-acyl), LPE, PI, PG, CL, PA, LPA, PS, LPS, Cer and CerPE were analysed in the negative ion mode. Each of the quantified lipid species accounted for more than 0.5% within its lipid class.

Lipids were identified by LipidXplorer software [57] by matching *m*/*z* of their monoisotopic peaks to the corresponding elemental composition constraints. Mass tolerance was 2 ppm and intensity threshold was set according to the noise level reported by the Xcalibur software (Thermo Scientific). The statistical analysis was performed according to Storey and Tibshirani, measuring statistical significance by calculating the *q*-values based on the concept of the false discovery rate, as described by Balogh *et al.* [58–60].

## Supplementary Material

Laurinyecz_et_al_SupplementaryFigures
