# Supplementary material for: Reduced expression of CDP-DAG synthase changes lipid composition and leads to male sterility in Drosophila
Source: Open Biol. 2016 Jan 20;6(1):150169. doi: 10.1098/rsob.150169 (PMC4736822; doi:10.1098/rsob.150169)
Supplement: Laurinyecz_et_al_SupplementaryFigures [file rsob150169supp1.pdf]

Supplementary Material

Figure S1. Post-meiotic phenotypes of the male sterile mutant and rescued *CdsA* testes. (A-D) Phenotypes of the male sterile *CdsA*^{EY08412} (A, B) and *CdsA*$^{UM\text{-}8246\text{-}3}$(C, D) testes. Confocal micrographs of dispersed actin cones in *CdsA*^{EY08412} (A) and *CdsA*$^{UM\text{-}8246\text{-}3}$(C) cysts. Phase contrast images of the whole testis with empty seminal vesicle (red line) in *CdsA*^{EY08412} (B) and *CdsA*$^{UM\text{-}8246\text{-}3}$(D). (E, G, I) Confocal micrographs of actin cones in *CdsA*^{ms1} revertant (E), CdsA-GFP; bamGal4, *CdsA*^{ms1} (G), and in β2tub-dPIS; *CdsA*^{ms1} (I) alleles. (F, H, J) Phase contrast images of whole testis with seminal vesicles (red line) and with matured, moving sperms (arrowhead) in *CdsA*^{ms1} revertant (F), CdsA-GFP; bamGal4, *CdsA*^{ms1}, (H), and in β2tub-dPIS; *CdsA*^{ms1} (J). Scale bars: 50 µm in A, C, E, G, I, and 100 µm in B, D, F, H, J.

Figure S2. Expression and localization of CdsA and the phenotype of the mutant and rescued *CdsA*^{ms1} testes. (A) Schematic representation of *CdsA-RA* and *CdsA-RB* transcripts with the localization of the P[EY08412] (at base 33 in the 5'UTR of *CdsA-RA*), P[UM-8246-3] (at base 41 in the 5'UTR of *CdsA-RA*), and P[CB0128] (at base 64 in the 5'UTR of *CdsA-RA*) elements, and the primers used in the quantitative RT-PCR. (B) Relative expression levels of *CdsA*-RA-RB and *CdsA*-RB in the testis of wild type and *CdsA*^{ms1} homozygotes were quantified by quantitative RT-PCR. Error bars indicate mean ± s.e.m. (C) Distribution of the number of cysts at various post-meiotic stages in wild type, *CdsA*^{ms1}*, CdsA*^{ms1} revertant, CdsA-GFP; *CdsA*^{ms1}, and β2tub-dPIS, *CdsA*^{ms1} testes, based on counting of elongated nuclei, individualization complexes (IC) (phalloidin staining), cystic bulges, and waste bags (Caspase-3 staining). Error bars indicate mean ± s.e.m. (D) Confocal micrograph of

spermatocytes expressing CdsA-GFP shows ER localization (green), where ER is labelled by anti-Calnexin99A (red) and DNA by DAPI (blue). Scale bar, 10 µm.

Figure S3. The assembly of individualization complex is not impaired in *CdsA*^{ms1} spermatids, but the individualization defects resulted in higher number of TUNEL positive structures. (A) Individual and merged fluorescent micrographs of wild type and *CdsA*^{ms1} cystic bulges stained for Lasp (green) and for F-actin (red). Scale bar, 10 µm. (B) Simultaneous DAPI staining (blue) and TUNEL assay (red) on spermatids. In the wild type there is no sign of cell death by TUNEL assay in the elongated cysts. Representative *CdsA*^{ms1} cyst contains TUNEL positive (red) structures in elongated spermatids. Scale bar, 40 µm. (C) Climbing test shows normal climbing velocity in mutants of *CdsA*^{ms1}. (D) Lipid class changes in the testes of wild type, *CdsA*^{ms1} mutant, and *CdsA*^{ms1} revertant line, measured by mass spectrometry.

Table S1. Molecular species profiles of lipids from adult testis of *Drosophila*. The levels are normalized to total membrane lipids. Values represent mean ± s.e.m, n=5; (*) wild type (WT) vs. *CdsA*^{ms1} $p<0.05$, $q<0.015$; (#) WT vs. β2tub-dPIS; *CdsA*^{ms1} $p<0.05$, $q<0.015$; ($) *CdsA*^{ms1} vs. β2tub-dPIS, *CdsA*^{ms1} $p<0.05$, $q<0.015$. Lipid values of dPIS, *CdsA*^{ms1} with significant compensatory-type changes are shown in bold.

Table S2. Sequence of primers used in cloning of *P{UASp-CdsA-GFP}*, *P{β2tub-dPIS}* plasmids and in quantitative RT-PCR experiments.

Figure S1.

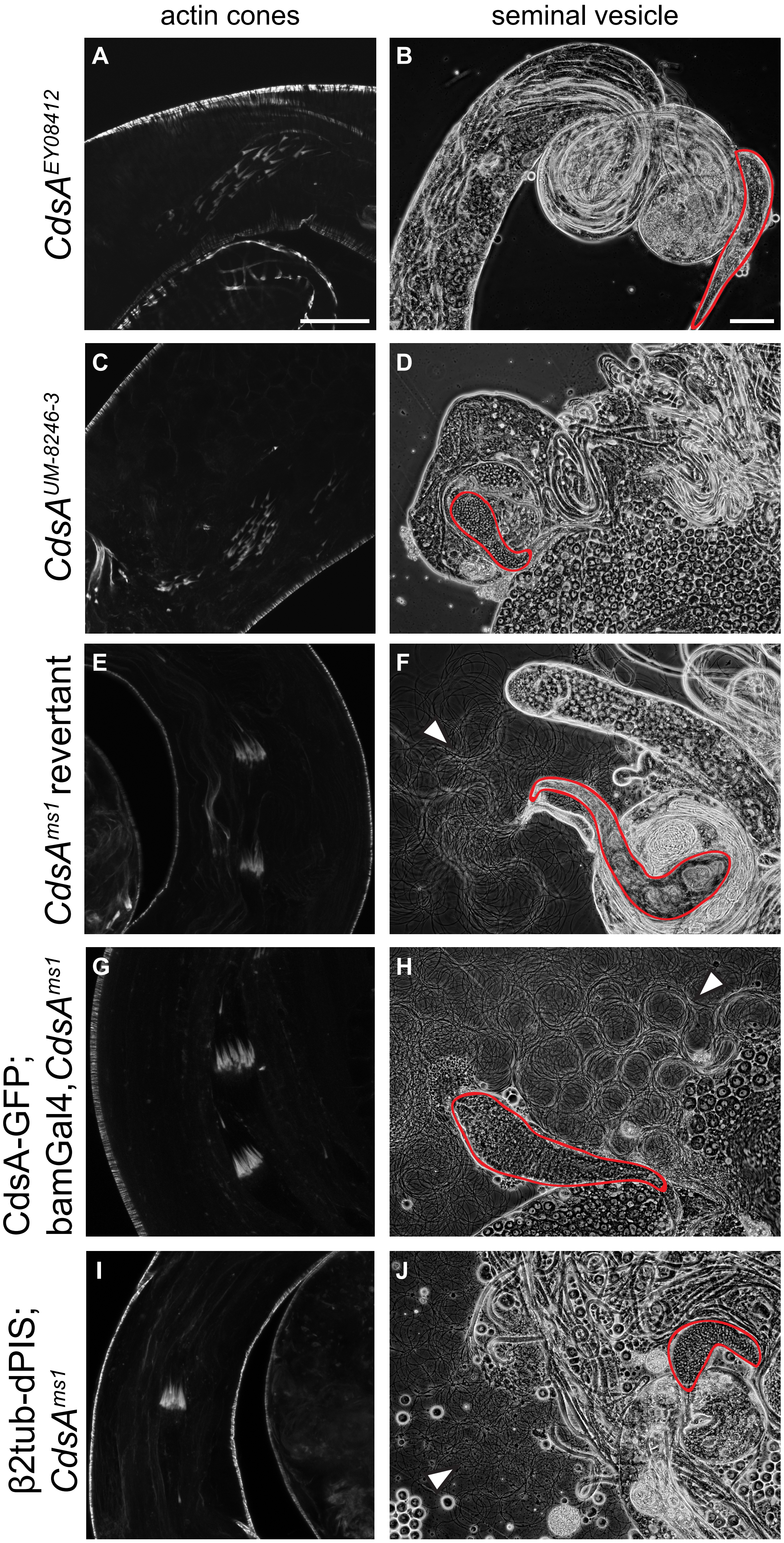
actin cones
seminal vesicle
A
B
C
D
E
F
G
H
I
J
$CdsA^{EY08412}$
$CdsA^{UM-8246-3}$
$CdsA^{ms1}$ revertant
CdsA-GFP; bamGal4,$CdsA^{ms1}$
β2tub-dPIS; $CdsA^{ms1}$

Figure S2.

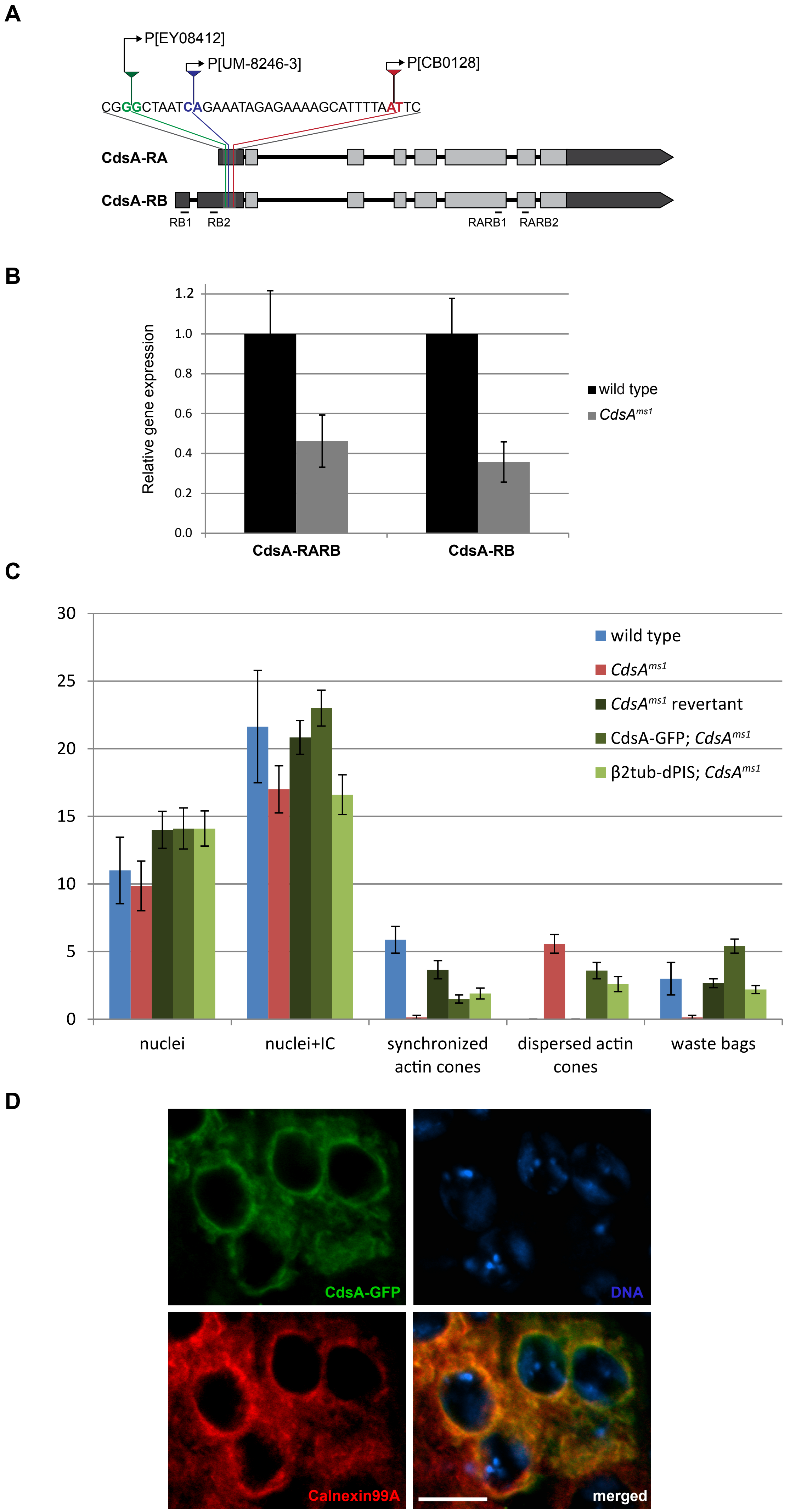
A
P[EY08412]
P[UM-8246-3]
P[CB0128]
CGGGCTAATCAGAAATAGAGAAAAGCATTTTAATTC
CdsA-RA
CdsA-RB
RB1
RB2
RARB1
RARB2
B
Relative gene expression
1.2
1.0
0.8
0.6
0.4
0.2
0.0
wild type
CdsA^ms1
CdsA-RARB
CdsA-RB
C
30
25
20
15
10
5
0
wild type
CdsA^ms1
CdsA^ms1 revertant
CdsA-GFP; CdsA^ms1
β2tub-dPIS; CdsA^ms1
nuclei
nuclei+IC
synchronized actin cones
dispersed actin cones
waste bags
D
CdsA-GFP
DNA
Calnexin99A
merged

Figure S3.

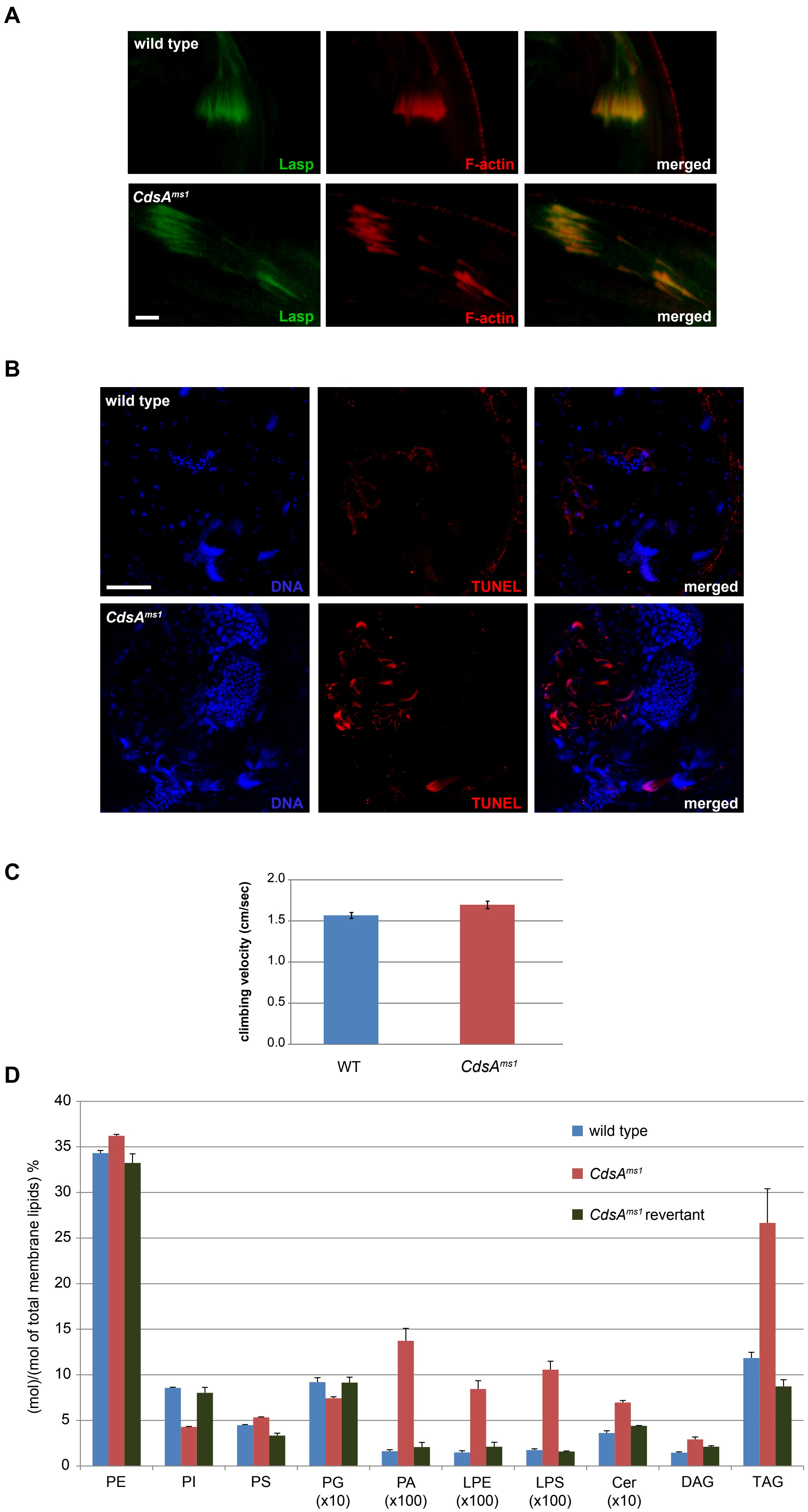
A
wild type
Lasp
F-actin
merged
CdsA^ms1
Lasp
F-actin
merged
B
wild type
DNA
TUNEL
merged
CdsA^ms1
DNA
TUNEL
merged
C
climbing velocity (cm/sec)
2.0
1.5
1.0
0.5
0.0
WT
CdsA^ms1
D
(mol)/(mol of total membrane lipids) %
40
35
30
25
20
15
10
5
0
wild type
CdsA^ms1
CdsA^ms1 revertant
PE
PI
PS
PG (x10)
PA (x100)
LPE (x100)
LPS (x100)
Cer (x10)
DAG
TAG

Table S1

(*) WT vs. *CdsA* ^{ms1} $p<0.05$, $q<0.015$

(#) WT vs. β2tub-dPIS, *CdsA* ^{ms1} $p<0.05$, $q<0.015$

($) *CdsA* ^{ms1} vs. β2tub-dPIS, *CdsA* ^{ms1} $p<0.05$, $q<0.015$

	**WT**	***CdsA*** ^{ms1}	**β2tub-dPIS, *CdsA*** ^{ms1}
PC [32:2]	1.182 ± 0.077	0.822 ± 0.019 *	**0.905 ± 0.028 # $**
PC [32:1]	2.281 ± 0.126	1.606 ± 0.029 *	**1.933 ± 0.026 # $**
PC [34:3]	2.325 ± 0.088	2.073 ± 0.030 *	2.141 ± 0.067
PC [34:2]	7.534 ± 0.106	6.680 ± 0.045 *	6.843 ± 0.078 #
PC [34:1]	6.755 ± 0.100	5.129 ± 0.141 *	5.456 ± 0.077 #
PC [36:4]	2.457 ± 0.036	4.095 ± 0.100 *	**3.429 ± 0.080 # $**
PC [36:3]	5.940 ± 0.165	7.739 ± 0.167 *	**6.866 ± 0.280 # $**
PC [36:2]	2.523 ± 0.036	3.340 ± 0.086 *	**2.743 ± 0.133 $**
PC [36:1]	0.226 ± 0.013	0.553 ± 0.040 *	**0.371 ± 0.047 # $**
PC	31.223 ± 0.304	32.038 ± 0.368	30.687 ± 0.483
PC-O [32:2]	0.213 ± 0.023	0.179 ± 0.007	0.119 ± 0.012 # $
PC-O [34:3]	1.705 ± 0.074	1.720 ± 0.054	1.728 ± 0.093
PC-O [34:2]	0.780 ± 0.019	1.039 ± 0.034 *	**0.649 ± 0.067 $**
PC-O [36:4]	0.657 ± 0.042	0.390 ± 0.019 *	0.366 ± 0.020 #
PC-O [36:3]	0.256 ± 0.010	0.212 ± 0.023	0.136 ± 0.010 # $
PC-O	3.611 ± 0.158	3.539 ± 0.109	2.999 ± 0.193 $
PE [30:1]	0.271 ± 0.018	0.252 ± 0.007	0.282 ± 0.041
PE [32:2]	0.629 ± 0.011	0.610 ± 0.014	0.656 ± 0.037
PE [32:1]	3.661 ± 0.086	3.519 ± 0.137	4.457 ± 0.137 # $
PE [34:3]	0.803 ± 0.034	0.882 ± 0.017	1.115 ± 0.041 # $
PE [34:2]	5.557 ± 0.025	6.330 ± 0.050 *	7.214 ± 0.177 # $
PE [34:1]	14.415 ± 0.088	14.351 ± 0.299	15.697 ± 0.156 # $
PE [36:4]	0.767 ± 0.032	0.897 ± 0.030 *	1.212 ± 0.053 # $
PE [36:3]	3.447 ± 0.146	3.723 ± 0.034	4.154 ± 0.089 # $
PE [36:2]	3.691 ± 0.104	3.996 ± 0.050 *	3.945 ± 0.088
PE [36:1]	0.626 ± 0.029	1.204 ± 0.067 *	**0.917 ± 0.052 # $**
PE [40:1]	0.463 ± 0.012	0.451 ± 0.005	0.479 ± 0.023
PE	34.331 ± 0.268	36.217 ± 0.147 *	40.129 ± 0.567 # $
LPE [16:1]	0.001 ± 0.000	0.006 ± 0.004	0.003 ± 0.001
LPE [16:0]	0.010 ± 0.001	0.018 ± 0.003	0.017 ± 0.002
LPE [18:2]	0.003 ± 0.001	0.009 ± 0.003	0.007 ± 0.002
LPE [22:0]	0.001 ± 0.000	0.051 ± 0.006 *	**0.014 ± 0.007 $**
LPE	0.015 ± 0.002	0.085 ± 0.014 *	**0.042 ± 0.003 # $**
PE-Pl [32:1]	0.603 ± 0.051	0.574 ± 0.029	0.357 ± 0.028 # $
PE-Pl [34:3]	0.337 ± 0.018	0.357 ± 0.010	0.766 ± 0.039 # $
PE-Pl [34:2]	5.225 ± 0.073	4.153 ± 0.105 *	**4.803 ± 0.158 $**

PE-Pl [34:1]	3.903 ± 0.052	4.711 ± 0.085 *	**2.844 ± 0.141 # $**
PE-Pl [36:3]	0.253 ± 0.005	0.138 ± 0.002 *	**0.165 ± 0.004 # $**
PE-Pl [36:2]	0.419 ± 0.012	0.427 ± 0.010	0.390 ± 0.013
PE-Pl [36:1]	0.244 ± 0.010	0.347 ± 0.006 *	**0.266 ± 0.006 $**
PE-Pl [38:3]	0.394 ± 0.010	0.382 ± 0.010	0.359 ± 0.008 #
PE-Pl [38:2]	0.522 ± 0.009	0.694 ± 0.014 *	**0.370 ± 0.017 # $**
PE-Pl [38:1]	0.186 ± 0.002	0.231 ± 0.010 *	**0.113 ± 0.006 # $**
PE-Pl	12.088 ± 0.141	12.014 ± 0.217	10.433 ± 0.307 # $
PI [32:1]	0.246 ± 0.013	0.175 ± 0.004 *	0.146 ± 0.009 # $
PI [34:3]	0.231 ± 0.006	0.164 ± 0.003 *	**0.205 ± 0.013 $**
PI [34:2]	1.940 ± 0.045	1.076 ± 0.030 *	**1.213 ± 0.036 # $**
PI [34:1]	2.413 ± 0.038	0.652 ± 0.021 *	0.656 ± 0.024 #
PI [36:4]	0.162 ± 0.005	0.129 ± 0.004 *	**0.165 ± 0.007 $**
PI [36:3]	1.355 ± 0.054	1.054 ± 0.019 *	1.086 ± 0.018 #
PI [36:2]	1.653 ± 0.004	0.889 ± 0.033 *	0.764 ± 0.057 #
PI [36:1]	0.567 ± 0.021	0.148 ± 0.006 *	0.157 ± 0.016 #
PI	8.568 ± 0.068	4.286 ± 0.050 *	4.393 ± 0.026 #
PS [32:1]	0.042 ± 0.001	0.053 ± 0.005	0.063 ± 0.002 #
PS [34:2]	0.164 ± 0.002	0.217 ± 0.003 *	0.246 ± 0.005 # $
PS [34:1]	0.288 ± 0.006	0.447 ± 0.012 *	0.496 ± 0.013 # $
PS [36:4]	0.064 ± 0.003	0.092 ± 0.003 *	0.107 ± 0.003 # $
PS [36:3]	0.336 ± 0.012	0.487 ± 0.015 *	0.515 ± 0.019 #
PS [36:2]	1.298 ± 0.045	1.438 ± 0.024 *	1.438 ± 0.052
PS [36:1]	0.191 ± 0.005	0.367 ± 0.019 *	**0.288 ± 0.016 # $**
PS [38:3]	0.020 ± 0.004	0.026 ± 0.003	0.035 ± 0.002 # $
PS [38:2]	0.194 ± 0.003	0.185 ± 0.001 *	0.180 ± 0.002 #
PS [38:1]	0.337 ± 0.007	0.303 ± 0.006 *	0.268 ± 0.006 # $
PS [40:2]	0.245 ± 0.003	0.322 ± 0.008 *	0.294 ± 0.016 #
PS [40:1]	0.930 ± 0.028	1.114 ± 0.025 *	1.035 ± 0.068
PS [42:2]	0.152 ± 0.006	0.134 ± 0.002 *	0.120 ± 0.004 # $
PS [42:1]	0.042 ± 0.003	0.028 ± 0.002 *	0.036 ± 0.004
PS [44:2]	0.169 ± 0.006	0.114 ± 0.002 *	**0.136 ± 0.005 # $**
PS	4.470 ± 0.068	5.326 ± 0.054 *	5.257 ± 0.126 #
LPS [20:0]	0.000 ± 0.000	0.006 ± 0.002 *	0.004 ± 0.002
LPS [22:0]	0.017 ± 0.002	0.098 ± 0.007 *	0.058 ± 0.025
LPS [24:1]	0.000 ± 0.000	0.002 ± 0.000 *	**0.000 ± 0.000 $**
LPS	0.017 ± 0.002	0.106 ± 0.009 *	0.062 ± 0.027
PG [30:2]	0.014 ± 0.004	0.008 ± 0.002	0.008 ± 0.003
PG [32:3]	0.032 ± 0.006	0.030 ± 0.006	0.025 ± 0.006
PG [32:1]	0.077 ± 0.008	0.066 ± 0.003	0.064 ± 0.002
PG [34:3]	0.068 ± 0.007	0.062 ± 0.002	0.077 ± 0.006 $
PG [34:2]	0.253 ± 0.010	0.211 ± 0.005 *	**0.247 ± 0.011 $**
PG [34:1]	0.314 ± 0.013	0.211 ± 0.005 *	0.213 ± 0.007 #
PG [36:4]	0.007 ± 0.001	0.011 ± 0.002	0.013 ± 0.001 #

PG [36:3]	0.088 ± 0.003	0.095 ± 0.004	0.098 ± 0.004
PG [36:2]	0.065 ± 0.000	0.050 ± 0.003 *	0.048 ± 0.003 #
PG	0.919 ± 0.050	0.744 ± 0.015 *	**0.792 ± 0.006 # $**
PA [32:1]	0.000 ± 0.000	0.002 ± 0.000 *	0.001 ± 0.000 #
PA [34:3]	0.000 ± 0.000	0.002 ± 0.000 *	0.001 ± 0.000 #
PA [34:2]	0.004 ± 0.000	0.027 ± 0.003 *	**0.013 ± 0.001 # $**
PA [34:1]	0.011 ± 0.001	0.061 ± 0.002 *	**0.038 ± 0.003 # $**
PA [36:3]	0.000 ± 0.000	0.008 ± 0.002 *	**0.003 ± 0.000 # $**
PA [36:2]	0.001 ± 0.000	0.027 ± 0.003 *	**0.004 ± 0.001 $**
PA [36:1]	0.000 ± 0.000	0.010 ± 0.002 *	**0.003 ± 0.001 # $**
PA	0.016 ± 0.002	0.137 ± 0.009 *	**0.063 ± 0.004 # $**
LPA [18:3]	0.001 ± 0.000	0.001 ± 0.000	0.001 ± 0.000
LPA [18:1]	0.000 ± 0.000	0.001 ± 0.000	0.000 ± 0.000
LPA [18:0]	0.001 ± 0.000	0.003 ± 0.001 *	0.002 ± 0.001
LPA [20:0]	0.000 ± 0.000	0.002 ± 0.000 *	0.001 ± 0.000
LPA	0.002 ± 0.000	0.007 ± 0.001 *	0.005 ± 0.001
CL [64:4]	0.013 ± 0.001	0.014 ± 0.002	0.015 ± 0.003
CL [66:5]	0.057 ± 0.003	0.048 ± 0.002	0.039 ± 0.002 # $
CL [68:7]	0.011 ± 0.001	0.011 ± 0.000	0.013 ± 0.001 $
CL [68:6]	0.166 ± 0.007	0.136 ± 0.004 *	0.101 ± 0.001 # $
CL [70:8]	0.027 ± 0.001	0.031 ± 0.000 *	0.040 ± 0.002 # $
CL [70:7]	0.274 ± 0.014	0.261 ± 0.009	0.215 ± 0.007 # $
CL [72:9]	0.034 ± 0.002	0.044 ± 0.002 *	0.069 ± 0.003 # $
CL [72:8]	0.217 ± 0.017	0.241 ± 0.016	0.251 ± 0.015
CL	0.800 ± 0.033	0.787 ± 0.030	0.742 ± 0.020
GlCer [32:1:2]	0.005 ± 0.001	0.009 ± 0.002	0.009 ± 0.004
GlCer [34:2:2]	0.028 ± 0.010	0.033 ± 0.021	0.017 ± 0.005
GlCer [34:1:2]	0.047 ± 0.002	0.064 ± 0.008	0.053 ± 0.011
GlCer [34:1:3]	0.014 ± 0.004	0.011 ± 0.002	0.009 ± 0.003
GlCer [36:2:2]	0.006 ± 0.001	0.013 ± 0.006	0.006 ± 0.000
GlCer [36:1:2]	0.024 ± 0.004	0.045 ± 0.013	0.037 ± 0.005
GlCer [38:2:2]	0.009 ± 0.005	0.012 ± 0.004	0.013 ± 0.005
GlCer [38:1:2]	0.014 ± 0.002	0.029 ± 0.007	0.020 ± 0.006
GlCer [40:1:2]	0.005 ± 0.003	0.010 ± 0.007	0.003 ± 0.002
GlCer	0.152 ± 0.006	0.226 ± 0.039	0.166 ± 0.016
Cer [32:1:2]	0.023 ± 0.001	0.030 ± 0.001 *	0.033 ± 0.002 #
Cer [34:2:2]	0.035 ± 0.003	0.036 ± 0.001	0.032 ± 0.002
Cer [34:1:2]	0.060 ± 0.003	0.090 ± 0.003 *	0.090 ± 0.004 #
Cer [34:1:3]	0.022 ± 0.002	0.030 ± 0.003	0.025 ± 0.005
Cer [36:2:2]	0.083 ± 0.003	0.102 ± 0.003 *	0.099 ± 0.004 #
Cer [36:1:2]	0.024 ± 0.002	0.045 ± 0.002 *	0.041 ± 0.003 #
Cer [36:1:3]	0.004 ± 0.001	0.038 ± 0.002 *	**0.020 ± 0.007 $**
Cer [38:2:2]	0.052 ± 0.003	0.082 ± 0.003 *	0.081 ± 0.004 #

Cer [38:1:3]	0.010 ± 0.004	0.115 ± 0.007 *	0.065 ± 0.025
Cer [40:2:2]	0.002 ± 0.000	0.005 ± 0.001 *	0.009 ± 0.001 # $
Cer [40:1:2]	0.003 ± 0.000	0.003 ± 0.001	0.005 ± 0.001
Cer [40:1:3]	0.015 ± 0.004	0.092 ± 0.005 *	0.060 ± 0.020
Cer [42:1:2]	0.012 ± 0.001	0.012 ± 0.002	0.015 ± 0.003
Cer [42:1:3]	0.018 ± 0.006	0.017 ± 0.006	0.030 ± 0.006
Cer	0.362 ± 0.025	0.697 ± 0.023 *	0.605 ± 0.058 #
CerPE [30:1:2]	0.054 ± 0.002	0.004 ± 0.004 *	0.029 ± 0.014
CerPE [32:2:2]	0.042 ± 0.001	0.029 ± 0.001 *	**0.033 ± 0.001 # $**
CerPE [34:2:2]	0.368 ± 0.019	0.329 ± 0.006	0.311 ± 0.011 #
CerPE [34:1:2]	0.704 ± 0.037	0.931 ± 0.008 *	**0.869 ± 0.015 # $**
CerPE [36:2:2]	1.306 ± 0.039	1.241 ± 0.043	1.168 ± 0.041
CerPE [36:1:2]	0.145 ± 0.009	0.292 ± 0.011 *	**0.244 ± 0.008 # $**
CerPE [38:3:2]	0.071 ± 0.003	0.062 ± 0.002 *	0.061 ± 0.001 #
CerPE [38:2:2]	0.679 ± 0.050	0.832 ± 0.024 *	0.822 ± 0.009 #
CerPE [40:2:2]	0.058 ± 0.003	0.071 ± 0.002 *	0.091 ± 0.002 # $
CerPE	3.426 ± 0.157	3.792 ± 0.090	3.627 ± 0.060
MAG [16:1]	0.005 ± 0.002	0.018 ± 0.005	0.016 ± 0.004
MAG [18:2]	0.099 ± 0.007	0.332 ± 0.028 *	**0.221 ± 0.034 # $**
MAG [18:1]	0.114 ± 0.031	0.406 ± 0.031 *	**0.278 ± 0.043 # $**
MAG [20:0]	0.217 ± 0.031	0.785 ± 0.059 *	**0.485 ± 0.104 $**
MAG [22:0]	0.005 ± 0.002	0.028 ± 0.007	0.021 ± 0.005
MAG	0.439 ± 0.069	1.570 ± 0.101 *	**1.022 ± 0.179 # $**
DAG [30:1]	0.075 ± 0.011	0.122 ± 0.009 *	0.096 ± 0.006
DAG [32:2]	0.087 ± 0.011	0.148 ± 0.015 *	0.135 ± 0.005 #
DAG [32:1]	0.209 ± 0.009	0.324 ± 0.025 *	0.273 ± 0.010 #
DAG [34:2]	0.164 ± 0.005	0.280 ± 0.016 *	0.247 ± 0.007 #
DAG [34:1]	0.225 ± 0.010	0.401 ± 0.027 *	0.331 ± 0.013 #
DAG [36:4]	0.035 ± 0.002	0.075 ± 0.008 *	0.056 ± 0.005 #
DAG [36:3]	0.087 ± 0.011	0.180 ± 0.013 *	**0.132 ± 0.003 # $**
DAG [36:2]	0.142 ± 0.009	0.278 ± 0.016 *	**0.215 ± 0.008 # $**
DAG [36:1]	0.160 ± 0.010	0.373 ± 0.047 *	**0.235 ± 0.012 # $**
DAG [38:2]	0.015 ± 0.004	0.097 ± 0.019 *	**0.035 ± 0.008 $**
DAG [38:1]	0.102 ± 0.022	0.276 ± 0.047 *	0.168 ± 0.020
DAG [40:1]	0.163 ± 0.017	0.363 ± 0.072	0.251 ± 0.033
DAG	1.464 ± 0.084	2.917 ± 0.271 *	**2.173 ± 0.017 # $**
TAG [42:2]	0.092 ± 0.006	0.257 ± 0.055	0.111 ± 0.039
TAG [42:1]	0.344 ± 0.014	0.830 ± 0.193	0.345 ± 0.099
TAG [42:0]	0.126 ± 0.018	0.455 ± 0.091 *	0.296 ± 0.071
TAG [44:2]	0.404 ± 0.016	0.936 ± 0.191	0.341 ± 0.085 $
TAG [44:1]	1.092 ± 0.045	2.191 ± 0.477	0.953 ± 0.248
TAG [46:3]	0.178 ± 0.007	0.386 ± 0.064	0.151 ± 0.039 $
TAG [46:2]	1.079 ± 0.046	2.304 ± 0.451	0.972 ± 0.230 $
TAG [46:1]	1.745 ± 0.083	3.784 ± 0.734	1.832 ± 0.389

TAG [48:3]	0.415 ± 0.016	0.840 ± 0.104 *	**0.475 ± 0.065 $**
TAG [48:2]	1.403 ± 0.076	3.065 ± 0.480 *	**1.569 ± 0.278 $**
TAG [48:1]	1.226 ± 0.068	3.076 ± 0.499 *	1.768 ± 0.322
TAG [50:4]	0.080 ± 0.008	0.186 ± 0.013 *	**0.137 ± 0.005 # $**
TAG [50:3]	0.359 ± 0.015	0.755 ± 0.061 *	**0.506 ± 0.039 # $**
TAG [50:2]	0.832 ± 0.040	1.899 ± 0.219 *	**1.142 ± 0.151 $**
TAG [50:1]	0.366 ± 0.021	0.820 ± 0.045 *	**0.627 ± 0.059 # $**
TAG [52:4]	0.148 ± 0.007	0.372 ± 0.011 *	**0.232 ± 0.018 # $**
TAG [52:3]	0.344 ± 0.019	0.798 ± 0.033 *	**0.496 ± 0.025 # $**
TAG [52:2]	0.385 ± 0.020	0.849 ± 0.027 *	**0.554 ± 0.033 # $**
TAG [54:5]	0.140 ± 0.014	0.334 ± 0.012 *	**0.174 ± 0.016 $**
TAG [54:4]	0.301 ± 0.023	0.716 ± 0.032 *	**0.343 ± 0.028 $**
TAG [54:3]	0.439 ± 0.028	0.905 ± 0.023 *	**0.487 ± 0.033 $**
TAG [54:2]	0.154 ± 0.011	0.376 ± 0.006 *	**0.199 ± 0.022 $**
TAG [54:1]	0.087 ± 0.015	0.248 ± 0.028 *	**0.154 ± 0.006 # $**
TAG [56:1]	0.102 ± 0.024	0.279 ± 0.045 *	0.191 ± 0.029
TAG	11.841 ± 0.621	26.661 ± 3.745 *	**14.057 ± 1.988 $**
Class			
PC	31.223 ± 0.304	32.038 ± 0.368	30.687 ± 0.483
PC-O	3.611 ± 0.158	3.539 ± 0.109	2.999 ± 0.193 $
PE	34.331 ± 0.268	36.217 ± 0.147 *	40.129 ± 0.567 # $
LPE	0.015 ± 0.002	0.085 ± 0.014 *	**0.042 ± 0.003 # $**
PE-Pl	12.088 ± 0.141	12.014 ± 0.217	10.433 ± 0.307 # $
PI	8.568 ± 0.068	4.286 ± 0.050 *	4.393 ± 0.026 #
PS	4.470 ± 0.068	5.326 ± 0.054 *	5.257 ± 0.126 #
LPS	0.017 ± 0.002	0.106 ± 0.009 *	0.062 ± 0.027
PG	0.919 ± 0.050	0.744 ± 0.015 *	**0.792 ± 0.006 # $**
PA	0.016 ± 0.002	0.137 ± 0.009 *	**0.063 ± 0.004 # $**
LPA	0.002 ± 0.000	0.007 ± 0.001 *	0.005 ± 0.001
CL	0.800 ± 0.033	0.787 ± 0.030	0.742 ± 0.020
GlCer	0.152 ± 0.006	0.226 ± 0.039	0.166 ± 0.016
Cer	0.362 ± 0.025	0.697 ± 0.023 *	0.605 ± 0.058 #
CerPE	3.426 ± 0.157	3.792 ± 0.090	3.627 ± 0.060
MAG	0.439 ± 0.069	1.570 ± 0.101 *	**1.022 ± 0.179 # $**
DAG	1.464 ± 0.084	2.917 ± 0.271 *	**2.173 ± 0.017 # $**
TAG	11.841 ± 0.621	26.661 ± 3.745 *	**14.057 ± 1.988 $**

Table S2.

**CdsA-attB1**	GGGGACAAGTTTGTACAAAAAAGCAGGCTTCATGGCCGAAGTGCGACGC
**CdsA-attB2**	GGGGACCACTTTGTACAAGAAAGCTGGGTTGGTTAACATGTCGCCCAAG
**dPIS-NotI**	ACTGCGGCGGCCGCAATGACAATTGCCGAGCAC
**dPIS-XbaI**	ACTGCGTCTAGATCACTCCACTTTCTTGCCGCTCA
**CdsA-RB1**	CCAGGGATGGCTGATATGGTC
**CdsA-RB2**	GTGGAGCAAAAGTGTTGGCAAG
**CdsA-RARB1**	GACGCATGACAATGTCCTGTG
**CdsA-RARB2**	GATCGAGTGCCAGATAAAGGG
**dPIS-Fw**	CGAGCACGATAACGTCTTCATC
**dPIS-Rev**	GCCGGAGATCACATAGTTGG
**rp49-Fw**	TCGTGAAGAAGCGCACCAAG
**rp49-Rev**	CTTGAAGCGGCGACGCAC
